# Epitranscriptomics: Toward A Better Understanding of RNA Modifications

**DOI:** 10.1016/j.gpb.2017.03.003

**Published:** 2017-05-19

**Authors:** Xushen Xiong, Chengqi Yi, Jinying Peng

**Affiliations:** 1 State Key Laboratory of Protein and Plant Gene Research, School of Life Sciences, Peking-Tsinghua Center for Life Sciences, Peking University, Beijing 100871, China; 2 Academy for Advanced Interdisciplinary Studies, Peking University, Beijing 100871, China; 3 Synthetic and Functional Biomolecules Center, College of Chemistry and Molecular Engineering, Peking University, Beijing 100871, China

Ever since the first RNA nucleoside modification was characterized in 1957 [1], over 100 distinct chemical modifications have been identified in RNA to date [2]. Most of these modifications were characterized in non-coding RNAs (ncRNAs), including tRNA, rRNA, and small nuclear RNA (snRNA) [3]. Studies in the past few decades have located various modifications in these ncRNAs and revealed their functional roles [3]. For instance, *N*^1^-methyladenosine (m^1^A), which is typically found at position 58 in the tRNA T-loop of eukaryotes, functions to stabilize tRNA tertiary structure [4] and affect translation by regulating the associations between tRNA and polysome [5]. Pseudouridine (Ψ) in snRNA can fine-tune branch site interactions and affect mRNA splicing [6], whereas Ψ in rRNA is required for binding to the internal ribosome entry site (IRES) and hence ensuring translational fidelity [7]. 5-methylcytidine (m^5^C) in tRNA can stabilize the secondary structure and maintain the anticodon stem-loop conformation [8], [9]. Many of these modifications in ncRNAs are conserved across different species, further indicating their biological significances [10].

Interestingly, *N*^6^-methyladenosine (m^6^A) was revealed to be also present in mRNA several decades ago [11], and its transcriptome-wide location has been mapped recently [12], [13] (Figure 1, Table 1). m^6^A modification in mRNA has been shown to play roles in gene expression regulation through multiple pathways, including affecting the stability, translation, splicing, and secondary structure of RNA molecules [54], [55], [56], [57], [58]. Besides m^6^A, several other modifications, including *N*^6^, 2′-*O*-dimethyladenosine (m^6^Am), inosine (I), m^5^C, Ψ, m^1^A, and 5-hydroxylmethylcytidine (hm^5^C), have also been revealed and mapped in mRNA and long ncRNA (lncRNA) [3], [58], [59] (Figure 1, Table 1). Recent studies have uncovered several potential biological roles for these mRNA modifications, such as Ψ-mediated translational read-through [50], m^1^A-associated translational regulation [51], and inosine-induced recoding [42] (Table 1). Taking together, studies on RNA modifications have led to the emergence of a new field, “epitranscriptomics”, which aims to identify modifications in transcriptome and unravel their regulatory roles in biological processes [60].

**Figure 1:**
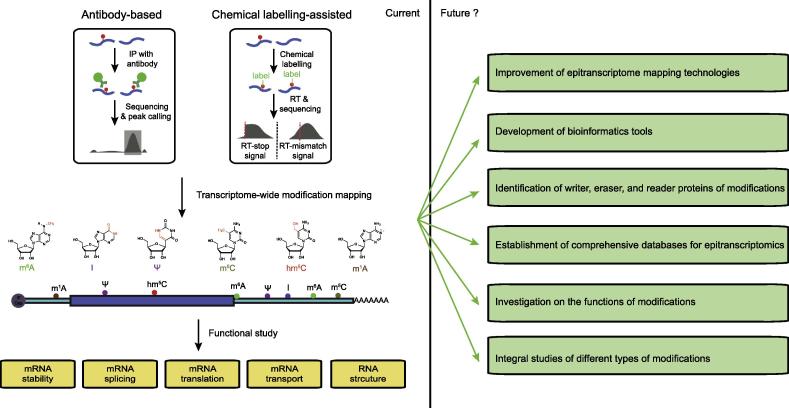
**Graphic summary illustrating the key content of current status and future directions of epitranscriptomics** The left panel shows the current status in the field of epitranscriptomics, including epitranscriptome mapping and identifying functional roles. Six types of known RNA modifications are indicated on the schematic plot of a typical mRNA, according to their preferential locations. The right panel shows the future directions of epitranscriptomics we discussed in the current article. m^6^A, *N*^6^-methyladenosine; I, inosine; Ψ, pseudouridine; m^5^C, 5-methylcytidine; hm^5^C, 5-hydroxylmethylcytidine; m^1^A, *N*^1^-methyladenosine.

**Table 1:** **Overview of biogenesis, abundance, distribution, and potential function of mRNA modifications in human**

**Modification**	**Biogenesis**	**mRNA**		
		**Abundance (in humans)**	**Distribution pattern**	**(Potential) function**
m^6^A	Methylated by the methyltransferase complex formed by METTL3, METTL14, and WTAP [14], [15], [16]	m^6^A/A: ∼0.1%–0.6% [17], [18], [19], [20], [21]	Enriched in 3′UTR and near stop codon [12], [13]; last exon [22]	Affecting RNA structure [23]; Reducing mRNA stability [24]; Enhancing mRNA translation [25]; Accelerating mRNA export [20]; Affecting pre-mRNA processing [26]

m^6^Am	Firstly methylated by 2′-*O*-MTase to form Am (CMTR1, CMTR2) [27] and then further methylated at *N*^6^ position by an unidentified nucleocytoplasmic methyltransferase [28]	m^6^Am/all nucleosides: ∼0.003% [29]	Exclusively TSS [30]	Enhancing mRNA stability [31]

m^5^C	Methylated by DNMT2 or NSUN2 [32], [33]	m^5^C/C: ∼0.025%–0.095% [34]	Enriched in 5′UTR and 3′UTR [32]	Inducing recoding [35]; Negatively affecting translation [36]

hm^5^C	Oxidized from m^5^C by TET [34], [37], [38]	hm^5^C/C: ∼0.001%–0.004% [32]; hm^5^C/m^5^C: ∼4% [32]	Not available	Associated with active translation [38]

I	Catalyzed by ADARs [39]	LC-MS/MS data not available	Mainly repetitive elements (*e.g.*, Alu and LINE) in UTRs and introns [40], [41]	Inducing recoding [42]; Influencing splice-site choice [43]; Affecting miRNA targeting capacity [43]

Ψ	Catalyzed by PUSs [44]	Ψ/U: ∼0.2%–0.4% [45]	Mainly CDS and 3′UTR [45], [46], [47]	Affecting RNA structure [48]; Enhancing mRNA stability [47]; Affecting mRNA translation [49]; Mediating mRNA read through [50]

m^1^A	Currently unknown for mRNA	m^1^A/A: ∼0.015%–0.054% [51], [52]	Enriched in 5′UTR and near start codon [51], [52]	Enhancing mRNA translation [51]; Affecting RNA structure [53]

*Note*: m^6^A, *N*^6^-methyladenosine; m^6^Am, *N*^6^,2′-*O*-dimethyladenosine; m^5^C, 5-methylcytidine; hm^5^C, 5-hydroxylmethylcytidine; I, inosine; Ψ, pseudouridine; m^1^A, *N*^1^-methyladenosine; METTL3, methyltransferase like 3; WTAP, Wilms’ tumor 1 associated protein; CMTR, Cap methyltransferase; DNMT2, DNA methyltransferase 2; NSUN2, NOP2/Sun RNA methyltransferase family member 2; ADAR, adenosine deaminase acting on RNA; PUS, pseudouridine synthase; TSS, transcriptional start site; LC-MS/MS, liquid chromatography-tandem mass spectrometry; LINE, long interspersed element.

Studies in the field of epitranscriptomics have revealed the functional importance of RNA modifications, yet many questions remain to be answered. While recent progresses in epitranscriptomics have been summarized in many excellent reviews [3], [54], [55], [56], [57], [58], [61], [62], we choose to discuss future research directions that could further advance our understanding of the field (Figure 1).

## Improvement of epitranscriptome mapping technology

Interest in the functional studies of RNA modifications has been revived in recent years. The majority of these studies are highly dependent on the existing transcriptome-wide mapping methods with the utilization of next-generation sequencing (NGS) technologies [59]. While the practicability of the current modification sequencing technologies has been demonstrated by the prosperities of recent NGS-based epitranscriptomics studies [3], [54], [55], [56], [57], [58], [61], [62], improvement of these technologies may further boost the studies of modifications in mRNA and lncRNA. Firstly, technologies that merely rely on the antibody-based RNA immunoprecipitation (RIP) cannot achieve single-base resolution [62]. Take the mapping technologies for hm^5^C and m^1^A for example, most of the modified sites can only be detected with resolutions of wider than 50 nucleotides [38], [51], [52]. Knowing the exact position of a modification, we are able to deduce its influence on the local RNA structure and investigate its downstream effects, such as alterations in protein binding [23]. Single-base resolution mapping technology also facilitates interrogation of the potential influence of mRNA modifications on biological processes like translational recoding and microRNA (miRNA) binding. Secondly, technologies that allow absolute stoichiometric quantification are still needed. In general, biologically essential modified sites, such as m^1^A in position 1322 of 28S rRNA and position 58 of tRNA^Met^ in humans, are modified with high stoichiometry [4], [63]. With the stoichiometric information for the modified sites, researchers will be able to evaluate their biological significances and investigate those highly-modified sites with higher priority. Of note, Molinie et al. developed a RIP-based method, namely m^6^A-level and isoform-characterization sequencing (m^6^A-LAIC-seq). m^6^A-LAIC-seq is capable of detecting m^6^A in a quantitative manner [29], indicating that quantitative information could be extracted by a RIP-based approach when an antibody with high specificity and sensitivity is available and the immunoprecipitation procedure is optimized enough for the full-length RNA pull-down. Thirdly, technologies for detecting the “co-existence” of modifications (either identical or different) in one transcript will be very helpful to reveal the potential interplays among different modifications. However, this can hardly be achieved with current NGS-based technologies, mainly due to the inevitable RNA fragmentation step in NGS sample preparation. Fourthly, current methods for transcriptome-wide modification mapping generally need more than 5 μg of mRNA [12], [33], [46], [47], [48], [52], [53]. If the required mRNA amount of starting materials can be reduced to the level of nanogram, it would enable the epitranscriptomic studies in the samples with limited availability (*e.g.*, clinical samples). Besides the widely-used NGS-based approaches, the rapidly-evolving third-generation sequencing (TGS) will, hopefully, provide a better approach for transcriptome-wide modification mapping. The single-molecule technologies, including PacBio’s single molecule real-time (SMRT) sequencing and Oxford Nanopore’s nanopore sequencing, have the potentials to directly detect modifications in the original nucleotide molecule [64], [65]. These technologies have already been applied to profile the modifications on DNA in mammalian and bacterial systems [66], [67]. Since the signal for every single nucleotide can be captured by TGS technologies, researchers would be able to detect all the modifications (either identical or different types) at single-base resolution within a RNA molecule. Moreover, as the procedure of PCR amplification, which may bring in bias [66], is no more required, quantitative information for the modified sites can also be retrieved. However, TGS technologies are still in their developing stages, thus their applicability in RNA modification detection remains to be seen.

## Development of bioinformatics tools

Most of the transcriptome-wide modification studies generate a large amount of sequencing data, bioinformatic tools are therefore greatly needed to aid the data analyses. Specifically, RIP-based transcriptome-wide mapping technologies are commonly used in epitranscriptomic studies. However, most of the currently available peak calling algorithms, such as zero-inflated negative binomial algorithm (ZINBA) [67], model-based analysis of ChIP-seq (MACS) [68], and HPeak [69] are programed for the data of ChIP-seq or DNase-seq [70], [71]. Different from such DNA sequencing data, RNA sequencing data generated from RIP assay would bear the bias of depletion in the two terminals due to the procedure of fragmentation [66]. Moreover, the alignment of epitranscriptomic data could be complicated by various isoforms transcribed from one identical genomic locus. Therefore, peak calling tools specifically designed for RIP data is necessitated. Of note, exomePeak is an RNA-seq based approach for detecting and visualizing the antibody-based epitranscriptome data, which has taken these issues into account [72]. However, for certain antibody-based technologies, the modifications would cause misincorporation or truncation during reverse transcription, providing additional information to elevate the resolutions [30], [51], [52], [73]. Thus, an optimized algorithm integrating both peak detection and reverse transcription signature capture could further facilitate the modification mapping analysis. In addition, several modifications, including m^6^A, Ψ, and m^1^A, would affect the Watson–Crick base-pairing, hence influencing the local RNA structure [23], [48], [53]. Given that RNA structure could affect the protein binding and alter the fate of RNA molecules [23], software for evaluating the local structure and energy changes caused by modifications would be useful as well.

## Identification of writer, eraser, and reader proteins of modifications

For modification studies, it is important to identify the enzymes responsible for the installation and removal of a modification (known as “writer” and “eraser”, respectively), as well as the modification binding proteins (“reader”). By manipulating the expression of the writer or eraser (if any) for a modification, we can observe the global change of the modification and further investigate the downstream biological effects. For example, many functional studies of m^6^A modification, including regulation on mRNA stability, translation efficiency, and exon inclusion, relied on manipulating the methyltransferases METTL3/METTL14/WTAP [55], [56], [57], [58], [59]. Of note, these regulatory roles of m^6^A were found to be mediated by its readers, mainly the YT521-B homology (YTH) domain-containing proteins [12], [55], [56], [57], [58], [59]. Thus, for other newly-mapped mRNA modifications besides m^6^A, identification of their writer, eraser, and reader proteins would be very important and helpful in exploring their functions, as well as revealing the underlying mechanisms. The initial discovery of m^6^A readers relies on the RNA pull-down assay using synthesized oligonucleotides containing m^6^A [12]. Such method may also be imitated to identify readers for other modifications. As for the screening of writer proteins, purification of the fractions that are necessary for modifying activity from the cell extract could be a practical approach. Enzymatic activity can be evaluated through *in vitro* incubation of an unmodified RNA oligonucleotide with different fractions from cell extract. Such strategy proved successful in discovering METTL3 (also known as MT-A70), one component of m^6^A writer [74]. In addition, modification enzymes may also be identified *de novo* through bioinformatic prediction based on the conservation of protein domains involved, followed by the experimental validation using assays like knock-down or overexpression [15]. However, for the modification like Ψ, which has multiple writers, functional study could be quite complicated due to the redundancy and potential interplay of the modifying enzymes. In the case of m^5^C, whose writer could target both mRNA and tRNA, it could be difficult to distinguish the effect caused by the modifications in mRNA or in tRNA. Under such circumstances, assays like photoactivatable ribonucleoside-enhanced crosslinking and immunoprecipitation (PAR-CLIP) or individual-nucleotide resolution CLIP (iCLIP) could be very useful to identify the global targets of a writer.

## Establishment of comprehensive database for epitranscriptomics

A comprehensive database for curating and sharing epitranscriptomic data should be established. The National Institutes of Health (NIH) has built the “Roadmap Epigenomics Mapping Consortium” to integrate the public resources for epigenomics studies [75]. This consortium also aims to standardize the experimental and computational procedures to close the gaps between different studies. Accordingly, as more important roles of RNA modifications are revealed and broader focuses are received for this area, a similar project or database should also be established for epitranscriptomics study in the near future. This would certainly help to facilitate and standardize the study of RNA modifications, hence accelerating the development of this area.

## Investigation on the functions of modifications

Since m^6^A is the first RNA modification being profiled transcriptome-wide with its writer, eraser, and reader identified, the functional roles of this methylation have received extensive attention in the field. Hopefully, besides m^6^A, other modifications in mRNA could also share the limelight. In fact, potential functions of a certain type of modification could be hinted by its distribution pattern. Take m^1^A for example, a prominent feature of highly-enriched distribution pattern in 5′UTRs and start codon has been revealed in both humans and mice [51], [52], suggesting that m^1^A may be involved in translational regulation. Besides, the intrinsic property of a modification would also provide us some clues for generating new hypotheses. Modifications occurring in the Watson–Crick edge, such as m^5^C and m^1^A, would affect the normal base pairing, hence altering the local RNA structure or causing recoding of the translated peptides [35], [53]. On the other hand, besides the widely-utilized cultured cells, animal or disease models that are deficient in writers, readers, or erasers also need to be established. These systems could facilitate the investigations on physiological roles of a particular modification, such as its relations to fertility, differentiation, and pathogenicity [16], [20], [76], [77], [78]. Hopefully, with the help of these model systems, we can achieve a deeper understanding of the molecular mechanism underlying the pathogenesis, and develop the therapies for the diseases associated with these RNA modifications. Furthermore, the dynamic pattern of a particular modification may have the potential to serve as a biomarker to monitor the status of the disease, provided that the correlation between a certain disease and a modification could be illustrated. In fact, DNA modifications have already been discovered as biomarkers for the early detection of cancer [79]. As m^6^A has been suggested to connect with cell differentiation and cancer formation [80], the possibility of mRNA modifications as biomarkers could be tested in the near future.

## Integral study of different types of modifications

The study of epitranscriptome could evolve toward an integral manner that comprehensively considers the effect of multiple types of modifications in the transcriptome. Most of the current studies in epitranscriptome are focused on a single particular modification. However, various modifications in RNA may interplay with each other, forming “networks” to modulate the physiological pathways. For instance, multiple types of modifications, including m^6^A, m^1^A, and Ψ, have been identified in *MALAT1* [23], [45], [51], [52], a nuclear speckle localized lncRNA; yet it remains unknown whether these different types of modifications would affect the installation of each other, or whether they would work together to maintain the structure or stability of *MALAT1*. Along with the development of transcriptome-wide mapping technologies in the foreseeable future (especially the TGS technologies), integral studies of the RNA modifications could come into play.

Collectively, the functional studies of m^6^A have brought an initial period of prosperity in the emerging field of epitranscriptomics; in the meantime, several other modifications have also been identified and mapped in mRNAs and lncRNAs. We envision that with more endeavor being made in this field, researchers will get a better understanding of RNA modifications and their functions in a more comprehensive way.

## Competing interests

The authors declare no competing financial interests.

## References

[1] Davis F.F., Allen F.W. (1957). Ribonucleic acids from yeast which contain a fifth nucleotide. J Biol Chem.

[2] Dunin-Horkawicz S., Czerwoniec A., Gajda M.J., Feder M., Grosjean H., Bujnicki J.M. (2006). MODOMICS: a database of RNA modification pathways. Nucleic Acids Res.

[3] Li S., Mason C.E. (2014). The pivotal regulatory landscape of RNA modifications. Annu Rev Genomics Hum Genet.

[4] Schevitz R.W., Podjarny A.D., Krishanmachari N., Hughes J.J., Sigler P.B., Sussman J.L. (1979). Crystal structure of a eukaryotic initiator tRNA. Nature.

[5] Saikia M., Fu Y., Pavon-Eternod M., He C., Pan T. (2010). Genome-wide analysis of *N*^1^-methyladenosine modification in human tRNAs. RNA.

[6] Yu A.T., Ge J., Yu Y.T. (2011). Pseudouridines in spliceosomal snRNAs. Protein Cell.

[7] Jack K., Bellodi C., Landry D.M., Niederer R.O., Meskauskas A., Musalgaonkar S. (2011). RRNA pseudouridylation defects affect ribosomal ligand binding and translational fidelity from yeast to human cells. Mol Cell.

[8] Squires J.E., Preiss T. (2010). Function and detection of 5-methylcytosine in eukaryotic RNA. Epigenomics.

[9] Motorin Y., Helm M. (2010). TRNA stabilization by modified nucleotides. Biochemistry.

[10] Machnicka M.A., Milanowska K., Osman Oglou O., Purta E., Kurkowska M., Olchowik A. (2012). MODOMICS: a database of RNA modification pathways–2013 update. Nucleic Acids Res.

[11] Desrosiers R., Friderici K., Rottman F. (1974). Identification of methylated nucleosides in messenger RNA from Novikoff hepatoma cells. Proc Natl Acad Sci U S A.

[12] Dominissini D., Moshitch-Moshkovitz S., Schwartz S., Salmon-Divon M., Ungar L., Osenberg S. (2012). Topology of the human and mouse m^6^A RNA methylomes revealed by m^6^A-seq. Nature.

[13] Meyer K.D., Saletore Y., Zumbo P., Elemento O., Mason C.E., Jaffrey S.R. (2012). Comprehensive analysis of mRNA methylation reveals enrichment in 3′UTRs and near stop codons. Cell.

[14] Bokar J.A., Rath-Shambaugh M.E., Ludwiczak R., Narayan P., Rottman F. (1994). Characterization and partial purification of mRNA *N*^6^-adenosine methyltransferase from HeLa cell nuclei. Internal mRNA methylation requires a multisubunit complex. J Biol Chem.

[15] Liu J., Yue Y., Han D., Wang X., Fu Y., Zhang L. (2014). A METTL3-METTL14 complex mediates mammalian nuclear RNA *N*^6^-adenosine methylation. Nat Chem Biol.

[16] Ping X.L., Sun B.F., Wang L., Xiao W., Yang X., Wang W.J. (2014). Mammalian WTAP is a regulatory subunit of the RNA *N*^6^-methyladenosine methyltransferase. Cell Res.

[17] Dubin D.T., Taylor R.H. (1975). The methylation state of poly A-containing messenger RNA from cultured hamster cells. Nucleic Acids Res.

[18] Wei C.M., Gershowitz A., Moss B. (1975). Methylated nucleotides block 5′ terminus of HeLa cell messenger RNA. Cell.

[19] Perry R.P., Kelley D.E., Friderici K., Rottman F. (1975). The methylated constituents of L cell messenger RNA: evidence for an unusual cluster at the 5′ terminus. Cell.

[20] Zheng G., Dahl J.A., Niu Y., Fedorcsak P., Huang C.M., Li C.J. (2013). ALKBH5 is a mammalian RNA demethylase that impacts RNA metabolism and mouse fertility. Mol Cell.

[21] Jia G., Fu Y., Zhao X., Dai Q., Zheng G., Yang Y. (2011). *N*^6^-methyladenosine in nuclear RNA is a major substrate of the obesity-associated FTO. Nat Chem Biol.

[22] Ke S., Alemu E.A., Mertens C., Gantman E.C., Fak J.J., Mele A. (2015). A majority of m^6^A residues are in the last exons, allowing the potential for 3′UTR regulation. Genes Dev.

[23] Liu N., Dai Q., Zheng G., He C., Parisien M., Pan T. (2015). *N*^6^-methyladenosine-dependent RNA structural switches regulate RNA-protein interactions. Nature.

[24] Wang X., Lu Z., Gomez A., Hon G.C., Yue Y., Han D. (2014). *N*^6^-methyladenosine-dependent regulation of messenger RNA stability. Nature.

[25] Wang X., Zhao B.S., Roundtree I.A., Lu Z., Han D., Ma H. (2015). *N*^6^-methyladenosine modulates messenger RNA translation efficiency. Cell.

[26] Xiao W., Adhikari S., Dahal U., Chen Y.S., Hao Y.J., Sun B.F. (2016). Nuclear m^6^A reader YTHDC1 regulates mRNA splicing. Mol Cell.

[27] Liu J., Jia G. (2014). Methylation modifications in eukaryotic messenger RNA. J Genet Genomics.

[28] Keith J.M., Ensinger M.J., Mose B. (1978). HeLa cell RNA (2’-*O*-methyladenosine-*N*^6^-)-methyltransferase specific for the capped 5′-end of messenger RNA. J Biol Chem.

[29] Molinie B., Wang J., Lim K.S., Hillebrand R., Lu Z., Van Wittenberghe N. (2016). M^6^A-LAIC-seq reveals the census and complexity of the m^6^A epitranscriptome. Nat Methods.

[30] Linder B., Grozhik A.V., Olarerin-George A.O., Meydan C., Mason C.E., Jaffrey S.R. (2015). Single-nucleotide-resolution mapping of m^6^A and m^6^Am throughout the transcriptome. Nat Methods.

[31] Mauer J., Luo X., Blanjoie A., Jiao X., Grozhik A.V., Patil D.P. (2017). Reversible methylation of m^6^Am in the 5′ cap controls mRNA stability. Nature.

[32] Squires J.E., Patel H.R., Nousch M., Sibbritt T., Humphreys D.T., Parker B.J. (2012). Widespread occurrence of 5-methylcytosine in human coding and non-coding RNA. Nucleic Acids Res.

[33] Hussain S., Sajini A.A., Blanco S., Dietmann S., Lombard P., Sugimoto Y. (2013). NSun2-mediated cytosine-5 methylation of vault noncoding RNA determines its processing into regulatory small RNAs. Cell Rep.

[34] Huber S.M., van Delft P., Mendil L., Bachman M., Smollett K., Werner F. (2015). Formation and abundance of 5-hydroxymethylcytosine in RNA. ChemBioChem.

[35] Hoernes T.P., Clementi N., Faserl K., Glasner H., Breuker K., Lindner H. (2015). Nucleotide modifications within bacterial messenger RNAs regulate their translation and are able to rewire the genetic code. Nucleic Acids Res.

[36] Meyer K.D., Patil D.P., Zhou J., Zinoviev A., Skabkin M.A., Elemento O. (2015). 5′UTR m^6^A promotes cap-independent translation. Cell.

[37] Fu L., Guerrero C.R., Zhong N., Amato N.J., Liu Y., Liu S. (2014). Tet-mediated formation of 5-hydroxymethylcytosine in RNA. J Am Chem Soc.

[38] Delatte B., Wang F., Ngoc L.V., Collignon E., Bonvin E., Deplus R. (2016). Transcriptome-wide distribution and function of RNA hydroxymethylcytosine. Science.

[39] Nishikura K. (2010). Functions and regulation of RNA editing by ADAR deaminases. Annu Rev Biochem.

[40] Levanon E.Y., Eisenberg E., Yelin R., Nemzer S., Hallegger M., Shemesh R. (2004). Systematic identification of abundant A-to-I editing sites in the human transcriptome. Nat Biotechol.

[41] Athanasiadis A., Rich A., Maas S. (2004). Widespread A-to-I RNA editing of Alu-containing mRNAs in the human transcriptome. PLoS Biol.

[42] Nishikura K. (2016). A-to-I editing of coding and non-coding RNAs by ADARs. Nat Rev Mol Cell Biol.

[43] Behm M., Öhman M. (2016). RNA editing: a contributor to neuronal dynamics in the mammalian brain. Trends Genet.

[44] Li X., Ma S., Yi C. (2016). Pseudouridine: the fifth RNA nucleotide with renewed interests. Curr Opin Chem Biol.

[45] Li X., Zhu P., Ma S., Song J., Bai J., Sun F. (2015). Chemical pulldown reveals dynamic pseudouridylation of the mammalian transcriptome. Nat Chem Biol.

[46] Carlile T.M., Rojas-Duran M.F., Zinshteyn B., Shin H., Bartoli K.M., Gilbert W.V. (2014). Pseudouridine profiling reveals regulated mRNA pseudouridylation in yeast and human cells. Nature.

[47] Schwartz S., Bernstein D.A., Mumbach M.R., Jovanovic M., Herbst R.H., León-Ricardo B.X. (2014). Transcriptome-wide mapping reveals widespread dynamic-regulated pseudouridylation of ncRNA and mRNA. Cell.

[48] Arnez J.G., Steitz T.A. (1994). Crystal structure of unmodified tRNA^Gln^ complexed with glutaminyl-tRNA synthetase and ATP suggests a possible role for pseudouridines in stabilization of RNA structure. Biochemistry.

[49] Fernández I.S., Ng C.L., Kelley A.C., Wu G., Yu Y.T., Ramakrishnan V. (2013). Unusual base pairing during the decoding of a stop codon by the ribosome. Nature.

[50] Karijolich J., Yu Y.T. (2011). Converting nonsense codons into sense codons by targeted pseudouridylation. Nature.

[51] Dominissini D., Nachtergaele S., Moshitch-Moshkovitz S., Peer E., Kol N., Ben-Haim M.S. (2016). The dynamic *N*^1^-methyladenosine methylome in eukaryotic messenger RNA. Nature.

[52] Li X., Xiong X., Wang K., Wang L., Shu X., Ma S. (2016). Transcriptome-wide mapping reveals reversible and dynamic *N*^1^-methyladenosine methylome. Nat Chem Biol.

[53] El Yacoubi B., Bailly M., de Crécy-Lagard V. (2012). Biosynthesis and function of posttranscriptional modifications of transfer RNAs. Annu Rev Genet.

[54] Niu Y., Zhao X., Wu Y.S., Li M.M., Wang X.J., Yang Y.G. (2013). *N*^6^-methyladenosine (m^6^A) in RNA: an old modification with a novel epigenetic function. Genomics Proteomics Bioinformatics.

[55] Meyer K.D., Jaffrey S.R. (2014). The dynamic epitranscriptome: *N*^6^-methyladenosine and gene expression control. Nat Rev Mol Cell Biol.

[56] Yue Y., Liu J., He C. (2015). RNA *N*^6^-methyladenosine methylation in post-transcriptional gene expression regulation. Genes Dev.

[57] Liu N., Pan T. (2016). *N*^6^-methyladenosine–encoded epitranscriptomics. Nat Struct Mol Biol.

[58] Gilbert W.V., Bell T.A., Schaening C. (2016). Messenger RNA modifications: form, distribution, and function. Science.

[59] Frye M., Jaffrey S.R., Pan T., Rechavi G., Suzuki T. (2016). RNA modifications: what have we learned and where are we headed?. Nat Rev Genet.

[60] Saletore Y., Meyer K., Korlach J., Vilfan I.D., Jaffrey S., Mason C.E. (2012). The birth of the Epitranscriptome: deciphering the function of RNA modifications. Genome Biol.

[61] Song J., Yi C. (2017). Chemical modifications to RNA: a new layer of gene expression regulation. ACS Chem Biol.

[62] Li X., Xiong X., Yi C. (2017). Epitranscriptome sequencing technologies: decoding RNA modifications. Nat Methods.

[63] Waku T., Nakajima Y., Yokoyama W., Nomura N., Kako K., Kobayashi A. (2016). NML-mediated rRNA base methylation links ribosomal subunit formation to cell proliferation in a p53-dependent manner. J Cell Sci.

[64] Eid J., Fehr A., Gray J., Luong K., Lyle J., Otto G. (2009). Real-time DNA sequencing from single polymerase molecules. Science.

[65] Clarke J., Wu H.C., Jayasinghe L., Patel A., Reid S., Bayley H. (2009). Continuous base identification for single-molecule nanopore DNA sequencing. Nat Nanotechnol.

[66] Van Dijk E.L., Jaszczyszyn Y., Thermes C. (2014). Library preparation methods for next-generation sequencing: tone down the bias. Exp Cell Res.

[67] Rashid N.U., Giresi P.G., Ibrahim J.G., Sun W., Lieb J.D. (2011). ZINBA integrates local covariates with DNA-seq data to identify broad and narrow regions of enrichment, even within amplified genomic regions. Genome Biol.

[68] Zhang Y., Liu T., Meyer C.A., Eeckhoute J., Johnson D.S., Bernstein B.E. (2008). Model-based analysis of ChIP-Seq (MACS). Genome Biol.

[69] Qin Z.S., Yu J., Shen J., Maher C.A., Hu M., Kalyana-Sundaram S. (2010). HPeak: an HMM-based algorithm for defining read-enriched regions in ChIP-Seq data. BMC Bioinformatics.

[70] Koohy H., Down T.A., Spivakov M., Hubbard T. (2014). A comparison of peak callers used for DNase-seq data. PLoS One.

[71] Wilbanks E.G., Facciotti M.T. (2010). Evaluation of algorithm performance in ChIP-seq peak detection. PLoS One.

[72] Meng J., Cui X., Rao M.K., Chen Y., Huang Y. (2013). Exome-based analysis for RNA epigenome sequencing data. Bioinformatics.

[73] Chen K., Lu Z., Wang X., Fu Y., Luo G.Z., Liu N. (2014). High-resolution *N*^6^-methyladenosine m^6^A map using photo-crosslinking-assisted m^6^A sequencing. Angew Chem Int Ed Engl.

[74] Bokar J.A., Shambaugh M.E., Polayes D., Matera A.G., Rottman F.M. (1997). Purification and cDNA cloning of the AdoMet-binding subunit of the human mRNA *N*^6^-adenosine-methyltransferase. RNA.

[75] Chadwick L.H., The N.I.H. (2012). Roadmap Epigenomics Program data resource. Epigenomics.

[76] Hussain S., Tuorto F., Menon S., Blanco S., Cox C., Flores J.V. (2013). The mouse cytosine-5 RNA methyltransferase NSun2 is a component of the chromatoid body and required for testis differentiation. Mol Cell Biol.

[77] Sarin L.P., Leidel S.A. (2014). Modify or die? – RNA modification defects in metazoans. RNA Biol.

[78] Geula S., Moshitch-Moshkovitz S., Dominissini D., Mansour A.A., Kol N., Salmon-Divon M. (2015). Stem cells. m^6^A mRNA methylation facilitates resolution of naïve pluripotency toward differentiation. Science.

[79] Wang J., Han X., Sun Y. (2017). DNA methylation signatures in circulating cell-free DNA as biomarkers for the early detection of cancer. Sci China Life Sci.

[80] Jaffrey S.R., Kharas M.G. (2017). Emerging links between m^6^A and misregulated mRNA methylation in cancer. Genome Med.

